# From Biomarker Discovery to Targeted Clinical Application: Addressing Translational Gaps in Early Cancer Detection

**DOI:** 10.3390/biomedicines14061292

**Published:** 2026-06-05

**Authors:** Mohamad Adam Bujang

**Affiliations:** 1 Clinical Research Centre, Sarawak General Hospital, Ministry of Health Malaysia, Kuching 93586, Sarawak, Malaysia; mohamadadambujang1980@gmail.com or adam@crc.gov.my; 2 Institute for Clinical Research, National Institutes of Health, Ministry of Health Malaysia, Shah Alam 40170, Selangor, Malaysia

**Keywords:** breast cancer, clinical validation, diagnostic biomarkers, early detection, screening, translational research, health equity

## Abstract

Despite extensive progress in breast cancer biomarker research, only a limited proportion of candidate biomarkers successfully transition from early discovery to clinically validated tools for targeted early detection. This article examines the key translational barriers that impede this progression across the biomarker development continuum. Five major gaps are identified: (i) discovery-to-clinical relevance, (ii) methodological and analytical validation, (iii) regulatory and administrative complexity, (iv) translational performance and real-world integration, and (v) equity and deployment challenges. Collectively, these gaps highlight limitations not only in scientific and methodological rigor but also in validation frameworks, regulatory alignment, implementation feasibility, and healthcare system equity. This article emphasizes that successful biomarker translation requires more than analytical validity. Finally, it requires a supportive ecosystem that enables effective breast cancer early detection strategies and improves population-level outcomes in breast cancer care.

## 1. Introduction

Early detection remains one of the most effective strategies for reducing breast cancer mortality [1,2]. The clinical rationale for early detection is grounded in the observation that breast cancer outcomes are strongly stage-dependent, with significantly improved survival when the disease is identified at an earlier and more treatable stage. Over the past several decades, population-based screening programs and imaging modalities have contributed to improved outcomes. However, limitations in achieving targeted accuracy and population coverage continue to constrain their overall effectiveness, particularly in specific subgroups such as younger women and those with dense breast tissue.

In parallel with advances in clinical screening, rapid progress in molecular biology, genomics, transcriptomics, and proteomics have substantially expanded the landscape of potential diagnostic tools. These developments have enabled the identification of numerous candidate biomarkers, including circulating tumor DNA, microRNAs, methylation markers, and protein expression signatures [3,4,5,6,7]. Such biomarkers offer the theoretical advantage of enabling earlier detection through minimally invasive sampling, often using blood-based or liquid biopsy approaches. This has generated strong interest in biomarker driven strategies as complementary or alternative approaches to conventional imaging-based screening. Despite this considerable expansion in discovery-phase research, the translational success of these biomarkers has remained limited. Only a small proportion of proposed candidates progress beyond early experimental validation and reach the level of evidence required for integration into routine clinical practice [8]. This discrepancy highlights a persistent and fundamental challenge in translational oncology, which is the gap between biomarker discovery and clinically meaningful application.

This translational gap is not attributable to a single factor but reflects multiple inter-connected challenges operating across the research pipeline. Such fragmentation contributes to inefficiencies in validation, duplication of research efforts, and delays in translation. As a result, many promising biomarkers remain confined to research settings without achieving meaningful clinical impact. Understanding these interconnected barriers is therefore essential for improving the efficiency, reliability, and clinical utility of biomarker development in breast cancer. A structured examination of these gaps can help clarify why translation remains slow despite scientific advances and can inform more effective strategies for future research design and implementation.

Hence, this paper systematically examines the key translational gaps across the biomarker development pipeline and discusses their implications for improving the pathway from discovery to targeted clinical application. By identifying and integrating these challenges, the aim is to support a more coherent and clinically oriented framework for biomarker research. Subsequently, this will enhance early detection strategies and patient outcomes in breast cancer.

## 2. Gap 1: Discovery to Clinical Relevance

A major limitation in breast cancer biomarker research lies in the disconnect between early discovery-phase findings and clinically meaningful outcomes. Although biomarker discovery has advanced rapidly, translation into real-world clinical benefit remains limited. Discovery studies are typically conducted in controlled environments using small, carefully selected, and biologically homogeneous populations [3,4,5,6]. These designs are valuable for hypothesis generation and early signal detection, but they inherently prioritize internal validity over external generalizability.

This creates a structural imbalance in biomarker development. Findings that perform well under controlled conditions often fail to maintain performance when exposed to the complexity of clinical populations. In real-world settings, patients present with heterogeneous disease biology influenced by age, ethnicity, comorbidities, hormonal status, genetic predisposition, and environmental exposures [9,10]. These factors interact in ways that are rarely captured in early-phase studies.

Examples of emerging breast cancer biomarkers include circulating microRNAs, methylation-based markers, and multi-protein panels, which have demonstrated promising findings in early discovery studies. However, these biomarkers are often identified in selected populations under controlled conditions and may capture signals specific to particular biological contexts. Consequently, findings observed during early discovery phases may not consistently translate into broader and more heterogeneous clinical populations.

A related challenge is the absence of universally applicable biomarkers capable of reflecting the biological diversity of breast cancer. Because breast cancer consists of multiple molecular and clinical subtypes, biomarkers identified during early discovery phases often capture signals specific to selected populations or disease contexts. As a result, biomarkers demonstrating strong performance within derivation cohorts may not maintain comparable utility across broader patient populations. This limitation highlights the need to move beyond a “one-size-fits-all” biomarker paradigm toward subtype-informed or integrated biomarker strategies that better account for disease complexity.

Another key conceptual issue arises when statistical significance is interpreted as clinical relevance. In high-dimensional molecular studies, where thousands of variables are tested simultaneously, the risk of false discovery is substantial. Even when corrections for multiple testing are applied, effect size inflation may persist due to selection bias and model overfitting, leading to overestimation of biomarker performance [11,12,13].

To bridge this gap, a shift in research orientation is required, moving from discovery-centric models toward clinically anchored validation frameworks. Taken together, these limitations reflect a structural feature of early-phase biomarker research, which is primarily optimized for detectability rather than clinical applicability. When such findings are prematurely interpreted as clinically actionable without rigorous validation in diverse and representative populations, a persistent translational gap emerges, hindering progress toward effective and targeted population screening. This emphasizes the importance of continued systematic investigation to ensure that promising biomarkers can progress toward meaningful clinical implementation.

## 3. Gap 2: Validation

Methodological and analytical validation form the foundation of reliable biomarker re-search in breast cancer. Both represent the early essential checkpoint in the translational pathway from discovery to clinical application. Methodological rigor ensures that studies are designed with appropriate validity, statistical robustness, and reproducibility, while analytical validation ensures that biomarker measurements accurately and consistently reflect the biological constructs they are intended to capture. Together, these components determine whether early scientific findings can be translated into clinically meaningful tools.

Despite significant advances in biomarker discovery, reproducibility remains a central challenge in translational oncology. Many candidate biomarkers that initially demonstrate strong diagnostic or prognostic performance fail to reproduce consistently across independent laboratories [9,10]. This phenomenon is not incidental but reflects systematic weaknesses in the early phases of biomarker development. One of the major contributors to this problem is heterogeneity in assay methodology. Even when studies investigate the same biomarker, differences in assay platforms, detection chemistry, antibody specificity, or sequencing depth can lead to substantially different outcomes. Technologies such as liquid biopsy, circulating tumor DNA (ctDNA), and methylation-based assays illustrate the importance of analytical validation. Variations in sequencing depth, detection thresholds, sample processing, and laboratory protocols may significantly influence biomarker measurements and contribute to inconsistent findings across studies. These inconsistencies are often underestimated during interpretation of results, yet they directly influence the stability of reported associations.

In addition, pre-analytical variability represents a major but frequently underreported source of error. Differences in sample collection procedures, time to processing, storage temperature, freeze–thaw cycles, and transport conditions all influence biomarker stability [14,15]. In breast cancer research, where circulating biomarkers such as DNA fragments or microRNAs are particularly sensitive to degradation, these factors can substantially distort measured concentrations. Measurement bias further complicates interpretation. Calibration differences across laboratories, batch effects during data processing, and variability in platform-specific sensitivity and specificity all contribute to systematic distortion of biomarker performance estimates [16,17]. These biases often remain undetected until external validation is attempted, at which point discrepancies become apparent. A further limitation relates to statistical design. Many biomarker studies rely on small sample sizes combined with high-dimensional data structures. This creates conditions where overfitting is common and effect sizes are frequently inflated. Without proper validation strategies, models may appear highly accurate in derivation cohorts but fail when applied to independent datasets [18,19].

In addition, prospective and randomized validation studies are also essential before broad implementation of biomarkers into screening or surveillance programs. While many candidate biomarkers demonstrate promising performance in retrospective or early-phase studies, such designs may not adequately capture real-world clinical complexity and long-term outcomes. Prospective studies allow evaluation under more representative clinical conditions, whereas randomized designs can provide stronger evidence regarding clinical effectiveness, patient outcomes, and potential unintended consequences. Generating evidence from such studies is critical to determine whether biomarker-based strategies provide meaningful benefits beyond existing approaches and can support safe and effective integration into routine clinical practice.

To address these limitations, methodological reform is required at multiple levels. This includes the establishment of consensus standards, rigorous cross-laboratory validation, integration of prospective and randomized study designs, and regulatory-aligned frameworks to ensure that promising biomarkers can reliably inform patient care and serve as effective tools for early cancer detection. Embedding these principles within the study design, rather than applying them retrospectively, is critical for strengthening analytical robustness and improving translational reliability. Without such improvements, biomarker research risks generating non-reproducible findings and inflated performance estimates that ultimately fail to progress into clinically meaningful applications.

## 4. Gap 3: Administrative Complexity

The translation of breast cancer biomarkers from discovery to clinical application is strongly influenced by regulatory and standardization frameworks that govern diagnostic development, validation, and approval. While these frameworks are essential for ensuring safety, effectiveness, and scientific integrity, differences in regulatory structures across jurisdictions remain a major barrier to efficient translation.

In the United States, two distinct but related regulatory pathways are relevant. The U.S. Food and Drug Administration (FDA) Biomarker Qualification Program provides a structured mechanism to qualify biomarkers for a defined context of use, particularly in drug development and regulatory science. In parallel, biomarker-based diagnostic tests intended for clinical use are regulated under the FDA medical device framework for in vitro diagnostics (IVDs), which requires demonstration of analytical validity, clinical validity, and, where applicable, clinical utility prior to approval or clearance [20,21].

In the European context, biomarker-related activities within drug development are primarily considered within the remit of the European Medicines Agency (EMA), particularly when used to support therapeutic evaluation and regulatory decision-making. However, diagnostic tests themselves are not regulated by the EMA. Instead, they fall under the European Union In Vitro Diagnostic Regulation (IVDR 2017/746), which governs the approval of diagnostic devices through a system involving notified bodies and CE marking. The IVDR requires a stepwise demonstration of analytical performance, clinical performance, and scientific validity, with increased emphasis on clinical evidence and post-market surveillance compared with previous regulatory frameworks.

Despite these structured systems, differences in regulatory requirements between the FDA and EU IVDR frameworks can lead to duplicative validation studies, inconsistent evidentiary standards, and delays in global adoption [22]. These challenges are further amplified for integrated diagnostic approaches combining imaging modalities with biomarker signatures, as each component may require separate validation and regulatory assessment. Consequently, promising biomarkers may progress at different rates across jurisdictions, limiting their timely and harmonized integration into clinical practice. Overall, while regulatory and standardization systems are designed to ensure rigor and patient safety, fragmentation across regulatory frameworks may inadvertently increase administrative complexity and slow the translation of biomarker innovations into targeted clinical applications.

## 5. Gap 4: Translational Performance and Real-World Integration

Although mammography remains the primary screening tool for breast cancer, its limitations in sensitivity, particularly among younger women and those with dense breast tissue, have driven interest in biomarker-based complementary approaches [23]. Biomarker-based strategies may be particularly valuable for women with dense breast tissue, where conventional imaging performance is reduced. Approaches integrating imaging findings with biomarker information and risk-adapted screening strategies have therefore emerged as potential methods to improve individualized early detection. However, demonstrating analytical validity is only the initial step in a much more complex translational process. A central challenge lies in ensuring that biomarkers perform consistently across diverse real-world clinical environments. Unlike controlled research settings, clinical environments vary widely in terms of laboratory infrastructure, operator expertise, sample handling practices, and patient population characteristics. These variations can significantly influence test performance and reproducibility.

In addition, population-based screening for breast cancer is inherently resource-intensive, requiring substantial investment in infrastructure, laboratory capacity, trained personnel, and sustained health system support. As biomarker research progresses from discovery toward targeted clinical application, it becomes increasingly clear that analytical validity alone is insufficient to ensure successful translation into routine practice. Instead, implementation must be evaluated within the context of real-world healthcare system constraints. Cost-effectiveness is therefore a critical consideration, particularly in large-scale screening programs where even modest per-test costs can translate into significant financial implications at the population level [24,25].

Another critical requirement is the balance between sensitivity and specificity. High sensitivity is essential for early detection of disease, particularly in screening contexts where missing early-stage cancer can have serious consequences. However, excessively high sensitivity without adequate specificity can lead to increased false positives, unnecessary biopsies, patient anxiety, and additional healthcare costs. Conversely, overly strict specificity thresholds may reduce early detection rates. Many biomarkers are initially validated in small, homogeneous populations, which limits understanding of their performance in real-world settings [26,27]. These early cohorts often lack representation of key demographic and clinical variables such as ethnicity, age distribution, comorbidities, and genetic variability. As a result, performance estimates may not generalize to broader populations.

Real-world examples further illustrate the translational gap in breast cancer biomarker development. Several circulating biomarkers, including protein-based signatures, circulating tumor DNA (ctDNA), and microRNA profiles, have demonstrated promising diagnostic performance in early discovery and retrospective studies; however, none have yet been successfully implemented for population-based breast cancer screening. Limitations include inconsistent reproducibility across cohorts, variability in assay platforms, and insufficient evidence of impact on clinically meaningful outcomes such as stage shift or mortality reduction. In contrast, genomic assays such as Oncotype DX, MammaPrint, and PAM50 (Prosigna) have achieved successful clinical adoption. However, these tests are primarily used for prognostic stratification and treatment decision-making rather than early cancer detection. This divergence highlights that while biomarker translation has been successful in therapeutic guidance, its application in population-level early detection remains limited.

Another translational challenge relates to the reliability of biomarker performance across clinical settings. Biomarkers that initially demonstrate promising diagnostic accuracy may not consistently maintain performance under routine conditions due to biological variability and differences in clinical context. Even modest reductions in specificity may substantially increase false-positive findings in large-scale screening programs, whereas reduced sensitivity may compromise early detection efforts. Therefore, sustained performance reliability across different populations and healthcare environments remains essential before biomarkers can support targeted clinical implementation.

Therefore, clinical translation of breast cancer biomarkers requires not only robust analytical and clinical performance but also meaningful real-world utility. Healthcare system readiness also influences adoption. For instance, systems with limited infrastructure or workforce capacity may struggle to implement complex biomarker-based screening strategies, regardless of their clinical potential. Overall, translational success depends not only on scientific validity but also on operational feasibility. Alignment between diagnostic innovation and healthcare system capacity is essential. Without such alignment, even promising biomarkers may remain underutilized in routine clinical practice.

On another note, overdiagnosis and unintended harms should also be considered when evaluating biomarker-based early detection strategies. Although increased sensitivity may improve the identification of early-stage disease, it may also detect indolent lesions or biologically low-risk abnormalities that would not have progressed to clinically significant disease during a patient’s lifetime. This may contribute to overdiagnosis, leading to unnecessary investigations, overtreatment, psychological distress, and additional healthcare burden. Therefore, biomarker development should not focus solely on maximizing diagnostic accuracy but also consider the balance between early detection benefits and potential downstream harms to ensure meaningful clinical utility.

An additional consideration is the evidentiary threshold required before biomarkers can be implemented for early detection in asymptomatic populations. Compared with diagnostic use in symptomatic or high-risk groups, population screening demands stronger evidence because even small inaccuracies can affect large numbers of individuals. Therefore, evaluation should go beyond analytical and clinical validity to demonstrate meaningful improvements in clinically relevant outcomes.

Meanwhile, prospective and, where feasible, randomized studies are essential to confirm performance in representative populations and assess clinical effectiveness and unintended consequences. Key outcomes include stage shift, cancer-specific mortality, false positives, over diagnosis, and cost-effectiveness, as diagnostic accuracy alone may not translate into population-level benefit. Hence, biomarker-based approaches should be interpreted in relation to established mammography screening, which has proven mortality benefit. Rather than serving solely as replacements, biomarkers may function as complementary or risk-adapted tools, particularly in populations such as women with dense breast tissue.

## 6. Gap 5: Equity and Deployment Challenges

Even when breast cancer biomarkers demonstrate strong analytical and clinical performance, their real-world deployment introduces complex ethical, equity, and systemic challenges that significantly influence their population-level effectiveness. A primary concern is unequal access to diagnostic technologies. Advanced biomarker assays are often concentrated in urban tertiary hospitals or high-income healthcare systems [28]. In contrast, rural and resource-limited settings may lack access to the same diagnostic innovations due to limited laboratory infrastructure, shortages of trained personnel, and restricted healthcare resources. These disparities may delay diagnosis and reduce opportunities for early detection among underserved populations. To improve equitable implementation, deployment strategies should include decentralized diagnostic networks, regional laboratory hubs, and integration of biomarker testing into existing public health screening infrastructure. Mobile screening initiatives and simplified point-of-care platforms may also help expand access in geographically underserved regions.

Affordability further compounds this issue. Many modern biomarker technologies, particularly those involving multi-omics profiling, next-generation sequencing, or integrated molecular panels, are expensive to implement at scale. Advanced molecular technologies, including complex methylation-based platforms and integrated biomarker panels, frequently require specialized laboratory infrastructure and technical expertise. Such requirements may limit implementation in resource-constrained settings and widen disparities in access to early detection innovations. In addition, reimbursement limitations and differences in healthcare financing systems may further restrict adoption of biomarker-based screening approaches, even when clinical utility is demonstrated. To support broader implementation, scalable low-cost assays, tiered deployment strategies, and reimbursement frameworks aligned with public health priorities may be required. Phased implementation models that prioritize high-risk populations or regions with limited screening access may also improve cost-effectiveness and operational feasibility.

The increasing integration of artificial intelligence and machine learning into biomarker interpretation introduces additional ethical concerns [29]. If training datasets are not sufficiently diverse or representative, predictive models may perform unevenly across population subgroups. This can result in reduced diagnostic accuracy for underrepresented populations, thereby reinforcing existing healthcare inequalities [30]. Addressing this challenge requires the development of representative multi-ethnic datasets, external validation across diverse populations, transparent reporting of model performance, and continuous algorithm auditing to monitor fairness and reproducibility. Regulatory oversight and standardized reporting frameworks may also help reduce bias and improve reliability across healthcare settings.

Importantly, these challenges arise primarily during the implementation and deployment phase rather than the discovery phase. Unlike methodological limitations, which can often be addressed through improved experimental design, equity and deployment challenges require coordinated action at the policy, healthcare system, and governance levels. Successful translation therefore depends not only on scientific validity but also on implementation strategies that ensure accessibility, affordability, and representativeness across diverse populations.

Addressing these challenges requires more than technical innovation alone. It requires deliberate policy action to ensure equitable distribution of diagnostic technologies, investment in healthcare infrastructure in underserved regions, development of inclusive datasets for algorithm training, and reimbursement policies that support sustainable implementation. Equitable deployment may also benefit from risk-adapted and resource-sensitive implementation frameworks that align biomarker strategies with local healthcare capacity. Without such alignment, even scientifically robust biomarkers may remain underutilized or inaccessible in routine clinical practice. In this context, equity becomes not only an ethical consideration but also a scientific, translational, and public health necessity for achieving meaningful population-level impact in early cancer detection.

## 7. What to Do? Strengthening the Research Ecosystem

The persistent failure of promising breast cancer biomarkers to reach routine clinical application is not solely a consequence of all the gaps mentioned previously in this paper. Overall, it also reflects a deeper systemic weakness in the research ecosystem itself. These previously described gaps, although distinct in nature, are ultimately interconnected manifestations of fragmented governance, limited strategic coordination, and insufficient alignment between research generation and health system needs.

A critical issue is the absence of long-term translational continuity within biomarker research programs. Much of the current research activity is driven by short-cycle academic outputs, where success is measured by publication rather than progression along the translational pathway. This creates a structural discontinuity in which discovery, validation, and implementation are treated as isolated activities rather than as components of a unified development pipeline. As a result, promising biomarkers frequently lose momentum after initial publication, with no sustained mechanism to drive them toward clinical maturation.

Closely related to this is the lack of strategic prioritization at the ecosystem level. Biomarker research is often dispersed across multiple institutions without coordinated prioritization of targets based on clinical burden, feasibility, or scalability. This leads to a proliferation of candidate biomarkers with limited comparative evaluation, creating inefficiency in resource allocation and diluting efforts that could otherwise be concentrated on high-potential candidates. In well-developed translational ecosystems, prioritization mechanisms are essential to filter early discoveries into structured development pipelines.

Another fundamental weakness lies in the limited integration between research production systems and health system absorptive capacity. While individual studies may address specific scientific questions, the broader ecosystem may fail to ensure that research outputs are aligned with the operational realities of healthcare delivery systems. This disconnect results in a surplus of “technically promising” biomarkers that are not strategically developed with downstream adoption pathways in mind. Consequently, translational failure is not only scientific but also organizational.

Furthermore, sustainability of research progression remains a critical concern. Biomarker development requires iterative refinement over extended periods, yet funding structures are frequently project-based and short-term. This inhibits continuity across development phases and discourages investment in long-horizon validation studies. Without stable funding architecture that supports sequential progression from discovery to implementation, translational stagnation becomes structurally embedded.

**Figure 1:**
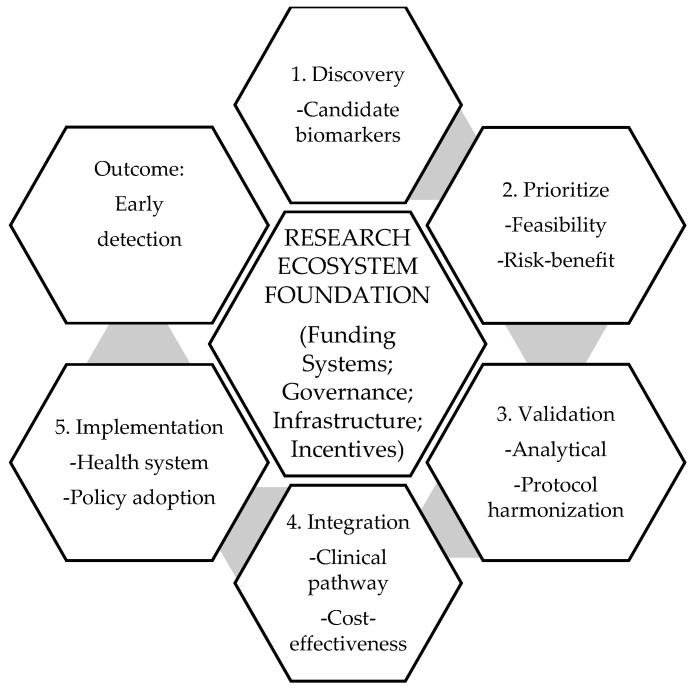
Research ecosystem foundation of biomarker research.

To address these systemic limitations, strengthening the research ecosystem requires a shift from fragmented research activity toward coordinated translational governance, as illustrated in Figure 1. This includes establishing structured national or regional biomarker development platforms that integrate discovery research (i.e., candidate biomarker) with clinical prioritization (i.e., feasibility and risk-benefit), validation pathways (i.e., analytical and protocol harmonization), integration (i.e., clinical pathway and cost-effectiveness), and implementation planning (i.e., health system and policy adoption). Such platforms should function as continuity mechanisms rather than isolated funding instruments.

Equally important is the development of incentive structures that reward translational progression rather than publication volume alone. Aligning academic recognition systems with downstream impact would encourage sustained development of biomarker candidates beyond initial discovery. In parallel, embedding clinical stakeholders and health system planners at early stages of research design would ensure that biomarker development is guided by real-world clinical demand and system feasibility.

Finally, strengthening ecosystem governance requires integration of data infrastructure and research coordination mechanisms. Promoting shared platforms for data integration, cross-institutional collaboration, and longitudinal tracking of biomarker candidates would reduce duplication and enable evidence consolidation. This would allow promising biomarkers to be systematically advanced rather than repeatedly rediscovered and abandoned at early stages.

## 8. Additional Note

Patient and patient organization engagement may also play an increasingly important role in guiding future biomarker development and implementation. Beyond scientific and technical performance, patient perspectives can help define clinically meaningful research priorities and determine which biomarker applications are relevant, acceptable, and useful in real-world settings. Meaningful engagement with patients throughout the research and translational process may therefore improve alignment between biomarker innovation and patient needs, supporting more patient-centered approaches across screening, surveillance, and broader disease management pathways.

This patient-centered paper is particularly relevant as the role of breast cancer biomarkers continues to expand beyond early detection. Emerging biomarker-based approaches are increasingly being explored for patient follow-up, detection of minimal residual disease, treatment monitoring, and disease surveillance. For example, circulating biomarkers and liquid biopsy technologies may enable dynamic assessment of disease status and facilitate earlier identification of recurrence or treatment response. As these applications evolve, integrating patient priorities becomes even more important, as translational challenges are not limited to screening alone but extend across the full continuum of breast cancer care.

Although the five translational gaps are presented as distinct domains, they should not be interpreted as isolated challenges. Rather, they represent interconnected stages within a broader translational continuum extending from biomarker discovery to targeted clinical implementation. Weaknesses arising in one stage may propagate downstream and influence subsequent phases, creating cumulative barriers that hinder successful translation into routine clinical practice.

For example, biomarkers identified during early discovery studies with limited clinical relevance or poor population representativeness (Gap 1) may enter validation pathways (Gap 2) with overly optimistic expectations, increasing the likelihood of reproducibility failures and inflated performance estimates. Even biomarkers that demonstrate satisfactory methodological and analytical validation may subsequently encounter regulatory and standardization barriers (Gap 3), delaying clinical approval and implementation. Furthermore, biomarkers that successfully navigate these stages may still experience reduced effectiveness when deployed within heterogeneous healthcare environments (Gap 4). Finally, successful implementation alone does not guarantee population-level impact if barriers related to accessibility, affordability, and equitable deployment remain unresolved (Gap 5).

Despite these interconnections, separate emphasis on each gap remains necessary because the underlying mechanisms and potential solutions differ substantially. Discovery-related challenges are largely biological and scientific in nature, validation barriers arise primarily from methodological and analytical limitations, administrative complexity reflects regulatory and governance processes, translational performance concerns involve operational and healthcare system factors, whereas deployment challenges are driven by broader ethical, socioeconomic, and equity considerations. Distinguishing these gaps enables targeted discussion and intervention strategies while preserving an integrated understanding of the broader translational pathway. Consequently, addressing these barriers requires not only isolated solutions but also coordinated efforts across multiple stages of biomarker development and implementation. Table 1 summarizes the translational gaps, implications, strategies, and potential impacts of the proposed actions.

## 9. Conclusions

Taken together, the five translational gaps outlined in this paper represent interconnected barriers that collectively limit the successful implementation of breast cancer biomarkers in clinical practice. These challenges indicate that translational failure is not driven by isolated weaknesses alone, but also by an underdeveloped and fragmented research ecosystem that fails to sustain continuity from discovery to clinical application. Strengthening this ecosystem is therefore fundamental to enabling meaningful progress. An integrated approach that combines methodological issues, coordinated validation, regulatory alignment, and system-level readiness is essential to accelerate the development of clinically actionable biomarkers. Such an approach will enhance early detection strategies and improve outcomes for patients at risk of breast cancer. On the other hand, although this discussion focuses on breast cancer, the challenges and proposed ecosystem-based solutions are broadly applicable to biomarker development and diagnostic research across a wide range of diseases.

## Figures and Tables

**Table 1:** Summary of Translational Gaps in Breast Cancer Biomarker Research.

Translational Gap	Clinical Implication	Recommended Strategy	Translational Impact
Gap 1: Discovery–Clinical Relevance	Limited generalizability of early findings	Validate in diverse populations; use clinically anchored frameworks	Improved clinical applicability
Gap 2: Validation	Poor reproducibility and assay variability	Standardization, cross-lab validation, prospective studies	Stronger analytical reliability
Gap 3: Administrative Complexity	Delayed and inconsistent regulatory approval	Regulatory harmonization and standardization	Faster clinical translation
Gap 4: Real-World Integration	Reduced effectiveness in routine practice	Prospective trials and cost-effectiveness evaluation	Better real-world performance
Gap 5: Equity & Deployment	Unequal access and algorithmic bias	Decentralized systems, affordable assays, fair AI models	More equitable implementation

## Data Availability

No new data were created or analyzed in this study>.

## Abbreviations

The following abbreviations are used in this manuscript:

DNA	deoxyribonucleic acid
microRNAs	Micro ribonucleic acids
EMA	European Medicines Agency
FDA	Food and Drug Administration
IVD	in vitro diagnostic
